# Control and prevention of infectious diseases from a One Health perspective

**DOI:** 10.1590/1678-4685-GMB-2020-0256

**Published:** 2021-01-29

**Authors:** Joel Henrique Ellwanger, Ana Beatriz Gorini da Veiga, Valéria de Lima Kaminski, Jacqueline María Valverde-Villegas, Abner Willian Quintino de Freitas, José Artur Bogo Chies

**Affiliations:** 1 Universidade Federal do Rio Grande do Sul - UFRGS, Departamento de Genética, Laboratório de Imunobiologia e Imunogenética, Porto Alegre, RS, Brazil.; 2 Universidade Federal do Rio Grande do Sul - UFRGS, Departamento de Genética, Programa de Pós-Graduação em Genética e Biologia Molecular - PPGBM, Porto Alegre, RS, Brazil.; 3 Universidade Federal de Ciências da Saúde de Porto Alegre - UFCSPA, Porto Alegre, RS, Brazil.; 4 Universidade Federal de São Paulo - UNIFESP, Instituto de Ciência e Tecnologia - ICT, Laboratório de Imunologia Aplicada, Programa de Pós-Graduação em Biotecnologia, São José dos Campos, SP, Brazil.; 5 Institut de Génétique Moléculaire de Montpellier (IGMM), Centre National de la Recherche Scientifique (CNRS), Laboratoire coopératif IGMM/ABIVAX, UMR 5535, Montpellier, France.; 6 Universidade Federal de Ciências da Saúde de Porto Alegre - UFCSPA, Programa de Pós-Graduação em Tecnologias da Informação e Gestão em Saúde, Porto Alegre, RS, Brazil.

**Keywords:** Global health, infection, One Health, pathogen, public health

## Abstract

The ongoing COVID-19 pandemic has caught the attention of the global community and rekindled the debate about our ability to prevent and manage outbreaks, epidemics, and pandemics. Many alternatives are suggested to address these urgent issues. Some of them are quite interesting, but with little practical application in the short or medium term. To realistically control infectious diseases, human, animal, and environmental factors need to be considered together, based on the One Health perspective. In this article, we highlight the most effective initiatives for the control and prevention of infectious diseases: vaccination; environmental sanitation; vector control; social programs that encourage a reduction in the population growth; control of urbanization; safe sex stimulation; testing; treatment of sexually and vertically transmitted infections; promotion of personal hygiene practices; food safety and proper nutrition; reduction of the human contact with wildlife and livestock; reduction of social inequalities; infectious disease surveillance; and biodiversity preservation. Subsequently, this article highlights the impacts of human genetics on susceptibility to infections and disease progression, using the SARS-CoV-2 infection as a study model. Finally, actions focused on mitigation of outbreaks and epidemics and the importance of conservation of ecosystems and translational ecology as public health strategies are also discussed.

## Introduction

Following the emergence of antibiotics and vaccination, many scientists and physicians believed that infectious diseases would cease to be major public health problems, and non-infectious chronic diseases would become more important in terms of public health, a phenomenon known as “epidemiological transition”. However, as a result of the emergence of new human pathogens, the re-emergence of many infections, and problems in controlling endemic diseases, infectious diseases remain major public health problems, along with non-infectious chronic diseases (McKeown, 2009; Bedford *et al.*, 2019). 

Considering disability-adjusted life years (DALYs) or number of infected individuals, the major infectious diseases on global scale are tuberculosis and other respiratory infections, HIV/AIDS and other sexually transmitted diseases, yellow fever, malaria, dengue and other mosquito-borne diseases, viral hepatitis, rabies, cholera, African trypanosomiasis, Chagas disease, leishmaniasis, cysticercosis/taeniasis, dracunculiasis, echinococcosis, cystic echinococcosis, trachoma, foodborne trematodiasis, lymphatic filariasis, onchocerciasis, schistosomiasis, ascariasis, trichuriasis, hookworm disease, Buruli ulcer, leprosy, and yaws (Bhutta *et al.*, 2014; Pigott *et al.*, 2015; GBD 2017 Disease and Injury Incidence and Prevalence Collaborators, 2018; Martins-Melo *et al.*, 2018). Although the impacts of these diseases are global, they affect countries disproportionately, being particularly important on populations living in tropical regions. Most of such diseases can be considered as neglected by governments, research institutions and the pharmaceutical industry, which aggravates the problem of infected individuals and countries where the diseases are endemic.

Urbanization, high population density, deforestation, climate changes, the growing interaction of humans with livestock and wild animals, the increase in the number and frequency of international travels, migratory flows, health system failures, social inequalities, and geopolitical conflicts are factors that contribute to the emergence and spread of new pathogens in human populations. As a consequence of the intensity of these factors in different parts of the world, outbreaks of emerging and re-emerging diseases are increasingly common worldwide, being considered “the new normal” in the field of infectious diseases (Bedford *et al.*, 2019; Ellwanger *et al.*, 2020a). In this context, Zika virus infection in the Americas (Faria *et al.*, 2017; Metsky *et al.*, 2017) and Ebola virus infection in Africa (Coltart *et al.*, 2017) became important in terms of public health in the last years. The ongoing Coronavirus Disease 2019 (COVID-19) pandemic (caused by Severe Acute Respiratory Syndrome Coronavirus 2 - SARS-CoV-2) exemplifies how a new human disease can emerge and spread globally in just a few months (Wu F *et al.*, 2020). Also, the COVID-19 pandemic showed that the capacity of countries to manage public health emergencies is quite heterogeneous, pointing out the need for coordinated and constant actions focused on global health.

**Figure 1 -:**
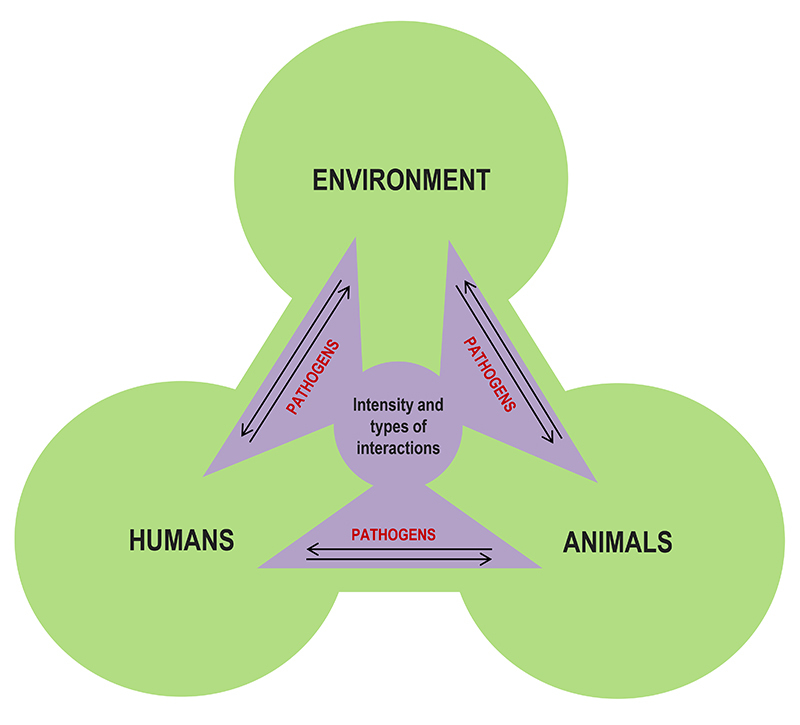
The One Health perspective. The intensity and types of interactions between humans, animals, and the environment determine the circulation of pathogens between these three actors. Therefore, these interactions can control infectious diseases or facilitate the emergence of outbreaks and epidemics, depending on how humans interact with the environment and other animals. The One Health perspective is used to understand the emergence and spread of infectious diseases and the best ways to control them. Adapted from Ellwanger (2019).

Each infectious disease has specific biological and ecological aspects, affecting geographic regions in different ways. Therefore, each disease requires specific control and preventive actions. However, there are effective “universal strategies” to prevent and mitigate infectious diseases in general, with very robust results, especially when such strategies consider human, environmental, animal and pathogen aspects in an integrated way from a One Health perspective (Calistri *et al.*, 2013; Rabinowitz and Conti, 2013; Cunningham *et al.*, 2017). According to this perspective, the intensity and types of interactions between humans, animals and the environment can control the spread of infectious diseases or facilitate the emergence of outbreaks and epidemics (Figure 1). One Health-based strategies are of high importance, especially in low- and middle-income countries, where infectious diseases are highly prevalent, and resources are limited. These classical initiatives must be part of both the discussion and practical actions of scientists and health professionals worldwide. Increasing efforts to implement and disseminate these actions, as well as to invest in technologies to improve them, is the best strategy for combating infectious diseases in a realistic approach (Ellwanger *et al.*, 2020a; Konda *et al.*, 2020; Mahrous *et al.*, 2020; Morens and Fauci, 2020; Wernli *et al.*, 2020). Thus, this review summarizes the most effective scientific-based initiatives for the control and prevention of infectious diseases aligned with the One Health perspective, in an updated manner.

In the first part of this article, the most effective actions for infectious disease control will be summarized, focused on the maintenance of adequate public health conditions. Subsequently, this article highlights the impacts of human genetics on susceptibility to infections and disease progression. For this purpose, the SARS-CoV-2 infection is used as a study model. Actions focused on mitigation of outbreaks that are useful to deal with public health emergencies are presented. This article also introduces the concept of conservation of ecosystems as a public health strategy. Finally, practical actions to avoid infectious diseases are suggested, considering the concept of translational ecology. This article can be considered a starting point for researchers and decision-makers to evaluate and select evidence-based actions to promote the control and prevention of infectious diseases in communities and research projects, increasing the impact of their actions on society.

## Actions for control and prevention of infectious diseases: an update

### Vaccination

In classic terms, vaccines are biological products that induce protective immunity against infections. Vaccines prevent ~2.5 million deaths each year. Currently, there are vaccines available internationally to prevent 26 infectious diseases. Also, there are other vaccines in the stage of development or already licensed. The positive impact that vaccines had on modern societies is undeniable. The maintenance of the health of human populations living in urban mega-clusters is only possible due to the prevention of infectious diseases spread, a situation also achieved through vaccination (Hodson, 2019; Piot *et al.*, 2019). Data from recent human history clearly show the positive impact of vaccination on our society. After causing about 400 million deaths in the 20th century, smallpox was eradicated in the 1970s after an international vaccination campaign coordinated by the World Health Organization (Nabel, 2013; Meyer *et al.*, 2020). Vaccines also played a fundamental role in the worldwide control of poliomyelitis, diphtheria, *Haemophilus influenzae*, measles, mumps, pertussis, rubella, and tetanus (Nabel, 2013; Orenstein and Ahmed, 2017).

Vaccines protect not only vaccinated individuals but also the community. Large-scale immunization reduces the exposure of non-vaccinated individuals to pathogens and interrupts or reduces the chain of disease transmission through the process known as “herd immunity”, characterized by the presence of numerous individuals immunized against a given pathogen. Also, under this strategy, immunodeficient individuals who cannot receive vaccines are protected. The best vaccines show, simultaneously, a long-lasting effect, cost-effectiveness, and safety, and are easy to administer in the population. All licensed vaccines are safe, once these products were subjected to rigorous safety tests before being made available to the population (Orenstein *et al.*, 2014; Orenstein and Ahmed, 2017; Piot *et al.*, 2019). 

Considering the broad benefits of vaccines in preventing infectious diseases, governments need to focus heavily on increasing vaccination coverage by conducting vaccination campaigns and establishing mechanisms to encourage vaccination, such as offering vaccines without cost to the citizens and ensuring easy access to vaccination services (e.g., offering free public transport on vaccination campaign days, or providing mobile vaccination stations to more peripheric communities). Private companies can contribute to vaccination efforts by allowing their employees to take their children to vaccination stations during working hours, without discounting their salaries. Moreover, public-private partnerships with the objective of reducing production costs and improving the distribution of vaccines are essential, especially in developing countries and during epidemic periods (Smith, 2000; Piot *et al.*, 2019; Vouking *et al.*, 2019).

Enabling the large-scale application of vaccines is often a task of high complexity, especially in remote regions of low-income countries. Several vaccines need to be stored and transported under refrigeration, requiring a stable supply of electricity to sustain the “cold-chain”, which is not always present in many remote regions. Also, the lack of qualified professionals to administer vaccines associated with the lack of medical supplies hamper vaccination campaigns. Therefore, it is necessary to develop thermostable and easy-to-apply vaccines, as well as to create effective means of transporting vaccines to remote regions (Orenstein *et al.*, 2014; Piot *et al.*, 2019). For example, unmanned aerial vehicles (drones) can be used to deliver vaccines to remote regions, where access by land is difficult (Haidari *et al.*, 2016; Rosser *et al.*, 2018).

Currently, one of the biggest challenges faced in the field of immunization is the anti-vaccine movement. Different society groups aligned with this movement disseminate non-scientific information through the media and internet questioning the safety of vaccines or the need for vaccination, fueling the vaccine hesitancy. These groups, together with the population’s lack of access to reliable information on vaccines, contribute to the decline in vaccine confidence (Piot *et al.*, 2019; Gostin *et al.*, 2020). Government efforts must be invested in the dissemination of information regarding the importance and safety of vaccines, clarifying the population’s doubts, and reducing the circulations of “fake news” and incorrect information concerning vaccines (Stahl *et al.*, 2016; Succi, 2018). Interestingly, the discussion about vaccines and vaccination coverage gained special attention during the recent SARS-CoV-2 pandemic. A huge number of articles and comments in the mass media both from the most traditional communication industry as well as electronic media, are raising awareness about this question and are pointing to vaccine development and unrestricted access to vaccination as essential steps in the control of this pandemic outbreak, thus rescuing the importance of science in our everyday life.

### Testing and diagnosis

The main infectious diseases currently affecting human populations are likely to be diagnosed through laboratory tests. The testing of infectious diseases and the identification of infected individuals among a specific population are the most critical initiatives for the success of policies focused on the control of infectious diseases. Testing affects the outcome of an infectious disease outbreak, basically, due to the interaction of five factors: 

First, testing (in this case, also diagnosis) is the initial step for the treatment of infected individuals. Second, individuals who know they are infected with a particular infectious agent can take measures to avoid its transmission. Third, large-scale testing enables the identification of the populations or communities most affected by a given infection, allowing mapping the areas where the problem is most pronounced and directing control and prevention policies more robustly to these target populations and specific locations. Four, virological surveillance trough the identification of viral strains (groups, subtypes, or recombinant strains) estimates recent infections and provides crucial data of viral epidemiology (Lot *et al.*, 2004). Finally, testing plays a crucial role in the surveillance of chronic infectious diseases and controlling outbreaks and epidemics, especially using point-of-care (POC) tests (Mabey *et al.*, 2004; Banoo *et al.*, 2006; Walensky *et al.*, 2007; Kozel and Burnham-Marusich, 2017).

More investments on POC tests associated with innovation and improvement of diagnostic tests will impact global health, especially in areas and populations with limited resources, where there is insufficient access to laboratories able to perform infectious disease testing (Abou Tayoun *et al.*, 2014). Tuaillon *et al.* (2020) highlighted the benefits of improving the diagnosis of infections by dried blood spot (DBP) sampling as a tool to increase the access to infectious disease diagnosis worldwide. Along with tests, reagents, consumables, equipment, maintenance, quality control assurance need to be also considered in these investments. Moreover, local manufacturing has to be improved to reach (in the medium and long term) independency from international suppliers. Dependence on a few international suppliers is very problematic, as noted in the current COVID-19 pandemic, in which many countries are having difficulty obtaining the necessary reagents and materials for SARS-CoV-2 testing.

It is important to emphasize that indiscriminately testing the general population may not be the best strategy in terms of surveillance and controlling infectious diseases from a public health perspective (not considering pandemic or epidemic situations, where large-scale testing is required). For example, conducting extensive tests for liver infections in the general population is likely to result in many negative and few positive results, at a high cost. On the other hand, targeting testing programs at individuals with risky behaviors is likely to result in the identification of a higher number of infected individuals at a lower cost (WHO, 2017a). For example, DBS sampling was used for the screening of HIV and hepatitis infections in key populations, such as intravenous drug users (McLeod *et al.*, 2014), sex workers (Shokoohi *et al.*, 2016), homeless people (Foroughi *et al.*, 2017), and men who have sex with men (Bogowicz *et al.*, 2016). In other words, it should be strategically evaluated which populations and areas such testing policies should be targeted more intensively. This direction must be based on risky behaviors and not on “risk groups”, avoiding stigmatization of specific portions of the population. 

Regarding sexually transmitted infections, diagnostic tests must be offered to any individual who expresses a personal interest in being tested. For example, in Brazil, HIV testing does not need a medical request and is freely available to the population through the Unified Health System (*Sistema Único de Saúde* - SUS) (Brasil, 2020). In brief, testing and diagnosis are the initial steps in a series of actions focused on the reduction of problems caused by infectious diseases. Taking the COVID-19 pandemic as an example, it is worth remembering the recent words of the WHO head Tedros Adhanom Ghebreyesus: “Our key message is: test, test, test”.

### Treatment

Treatment is the next expected step after an infectious disease is diagnosed. The treatment of infectious diseases has a pivotal effect on the life quality of infected individuals, reducing unwanted impacts of the disease on social and economic aspects, along with the avoidance of possible co-infection-related complications for the patient, such as for those co-infected with HIV and hepatitis C virus (Da Silva *et al.*, 2014; Valverde-Villegas *et al.*, 2017a). The treatment of infections is also essential for controlling certain diseases at the population level. For example, Cohen *et al.* (2011, 2016) showed that HIV-infected individuals under antiretroviral therapy and with undetectable viral load do not transmit the virus through sexual contact. This finding supported the “treatment as prevention” concept, which leads to the global U = U (undetectable = uninfectious) campaign (Cohen *et al.*, 2020). Also, the treatment of HIV-positive women has a very important impact on the reduction of mother-to-children infection, especially in low-income countries where HIV infection rates are high (Nagot *et al.*, 2016). Therefore, treating infections reduces the burden of infectious diseases and, considering HIV infection, prevents new cases of infections.

### Development of new antimicrobial drugs

Antimicrobial drugs are one of the main tools that humanity has to treat numerous infectious diseases and includes antibiotics, antivirals, antifungals, and antiparasitic agents. Among antimicrobial drugs, antibiotics have a prominent role due to their extensive use worldwide and the variety of therapeutic classes (e.g., β-Lactams, Tetracyclines, Rifamycins, Macrolides, Streptogramins, Quinolones), being used to treat several bacterial diseases, such as tuberculosis, gonorrhea, and leprosy. Humanity has benefited dramatically from the use of antibiotics, which have reduced both morbidity and mortality from bacterial infections. However, the indiscriminate and often inappropriate use of antibiotics in humans and other animals associated with the disposal of these drugs in the ecosystems has resulted in the selection of multi-resistant microorganisms. Antimicrobial resistance is currently one of the biggest problems in the field of infectious diseases. There is an urgent need to develop new antimicrobial drugs to be used in the treatment of infections by multidrug-resistant strains (Bronzwaer *et al.*, 2002; Coates *et al.*, 2002; Tenover, 2006; Morgan *et al.*, 2011; Piddock, 2012; Marston *et al.*, 2016). The benefits offered to human populations through the use of antimicrobial drugs are enormous and should not be overlooked by governments, research institutions, and the pharmaceutical industry. Although the development of new antimicrobial drugs is crucial for continued control of infectious diseases worldwide, the wide range of antimicrobial agents already available is an asset in the hands of researchers and the medical staff, enabling drug repurposing strategies. Nevertheless, the COVID-19 pandemic is teaching us that this resource should be used with extreme caution. Antimicrobials traditionally used to treat specific infections can be either effective or even deleterious when targeting new infectious agents in different contexts (Kandeel *et al.*, 2020; Mallhi *et al.*, 2020; Martinez, 2020; Senanayake, 2020).

### Reduction of contact with wildlife and livestock

Between 58-75% of infectious diseases that affect humans are originated from microorganisms hosted in non-human animals. In other words, the majority of human infectious diseases have a zoonotic origin (Taylor *et al.*, 2001; Woolhouse and Gowtage-Sequeria, 2005; Jones *et al.*, 2008; Ellwanger *et al.*, 2020a). The intense and close contact with wild animals creates opportunities for the emergence of new infectious diseases in humans since it allows the insertion of new pathogens in the human population (Wolfe *et al.*, 2005; Nava *et al.*, 2017). The transposition of pathogens between species is a complex process called zoonotic spillover. Such process depends on ecological factors and genetic characteristics of the hosts (humans and other species, including domestic and wild animals) and pathogens. The frequency and intensity of the contact between humans and other animals increase the chance of spillover events between these species and the emergence of new infectious diseases in humans. If the pathogen that was introduced into the human population is faced with favorable conditions for its replication and spreading, outbreaks or epidemics may occur (Morse, 1995; Plowright *et al.*, 2017; Ellwanger and Chies, 2018a).

The handling and consumption of meat, blood, and offal from wild animals (bushmeat/wildmeat) are very common in different regions worldwide, mainly in Asia and Africa, although in other regions of the world the bushmeat consumption is also very intense, as in Brazil (Mendonça *et al.*, 2016; Ripple *et al.*, 2016; Ordaz-Németh *et al.*, 2017). It is well known that this practice has facilitated the introduction of different pathogens in the human population, including HIV (Gao *et al.*, 1999; Hahn *et al.*, 2000). 

The Severe Acute Respiratory Syndrome Coronavirus (SARS-CoV) emerged in China in 2002-2003, and the Middle East Respiratory Syndrome Coronavirus (MERS-CoV) emerged in the Middle East in 2012, both as a result of the close interaction of humans with animals (Kock *et al.*, 2020). Bats are the natural hosts of SARS-CoV and MERS-CoV, whereas palm civets were the intermediate host for SARS-CoV, and camels were the intermediate host for MERS-CoV (Salata *et al.*, 2019; Xu, 2020). Again, it is very likely that the bushmeat trade contributed to the emergence of the recent human SARS-CoV-2 (COVID-19) pandemic (Li J *et al.*, 2020). Bats (Zhou P *et al.*, 2020) and pangolins (*Manis javanica*) (Lam *et al.*, 2020; Zhang *et al.*, 2020) host SARS-CoV-2-related coronaviruses. Hence, it is believed that bats probably served as natural reservoirs hosts of the virus and another species (pangolins or another animal species not yet identified) potentially served as an intermediate host, in which the virus may have evolved, ultimately acquiring characteristics that allowed this pathogen to infect humans and spread among the human population. However, the actual roles of these animal species in the emergence of SARS-CoV-2 are still under debate (Zhang and Holmes, 2020). In this context, the role of recombination of viruses from different species (bat and pangolin) in the emergence of the human SARS-CoV-2 is a possibility already suggested (Li X *et al.*, 2020). However, this aspect is still controversial and remains a matter of investigation (Boni *et al.*, 2020).

Considering the plethora of pathogens in wild animals and the intense interaction of humans with these animals, spillover events resulting in outbreaks and epidemics will repeatedly happen in human history. Thus, the way to reduce these events is to limit the interaction of humans with wild animals, mainly with mammals and birds (known as critical sources of zoonotic diseases). A compelling and cost-effective way to achieve this goal is to limit the wildlife trade and wildlife-derived products, especially in countries where bushmeat consumption endangers wild species (Kock *et al.*, 2020; Li J *et al.*, 2020; Zhang and Holmes, 2020).

Besides bushmeat, the trade of exotic animals to be used as pets also narrows the barriers between humans and exotic pathogens. Thus, the trade of exotic animals must be combated intensely. In addition, it is essential to make exotic pet owners and professionals who work in direct contact with animals (e.g., biologists, veterinarians, animal keepers) aware of the risks related to zoonosis and emerging diseases, potentially minimizing the risks of infections and spillover events (Chomel *et al.*, 2007; Rabinowitz and Conti, 2013). 

Revisiting the Nipah virus and avian influenza (H5N1 virus) cases, Morse *et al.* (2012) discuss the apparent opposing forces of economic development and public health. As conciliation alternatives, the authors proposed the creation of financial incentives for industries to reduce their environmental impacts, more secure food supply chains as an alternative to bushmeat trade, as well as more intensive surveillance of livestock in boundary areas with intense human-animal contact (Morse *et al.*, 2012). 

Human contact with livestock animals also facilitates the emergence of new human diseases, since numerous animals (and their pathogens) come into contact daily with humans whose work involves handling these animals. Of note, swine are prone to infection by avian and human influenza viruses, which allows the mixing of genetic information from different, but closely related, viral species (a phenomenon called genetic reassortment). This process facilitates the generation of new viral strains, including pandemic influenza viruses. As a consequence of this process, swine are considered “mixing vessels” for influenza viruses (Castrucci *et al.*, 1993; Zhou *et al.*, 1999; Brown, 2001; Ma *et al.*, 2009). 

In brief, new human infectious diseases are usually caused by pathogens that originally circulated only in wild animals and, after a spillover event, also infect and trigger diseases in humans (Taylor *et al.*, 2001; Jones *et al.*, 2008). These precepts are valid and easy to understand. However, allegations that wild animals, especially exotic species, are the causative agents of the emergence of new human diseases are common and incorrect. Non-human animals are actually the *source* of new human pathogens, but not the *cause* of the emergence of new diseases. The factors involved in the “jump” of pathogens from non-human animals to humans are generally associated with cultural and economic human practices, such as animal confinement for meat production, hunting, and deforestation. These practices intensify human contact with several animal species and with the wide range of pathogens hosted in such species, facilitating spillover events (Daszak *et al.*, 2000; Wolfe *et al.*, 2005; Espinosa *et al.*, 2020).

Taken together, reducing and controlling human contact with wild species and livestock animals is an important initiative to minimize the chances of the emergence of new infectious diseases in the human population. In this context, the requirement to comply with strict sanitary rules for meat trading needs to be extended to all meat trading locations, including popular and traditional markets (Daszak *et al.*, 2020; Dobson *et al.*, 2020). The probable link between the emergence of SARS-CoV-2 and a wet market in Wuhan city (Hubei province, central China) (Whitworth, 2020) clearly demonstrates the needs for the actions mentioned above.

### Biodiversity preservation and containment of climate change

The behavior and interactions of humans and non-human animals alter the abundance, diversity, and distribution of microorganisms in the environment. Similarly, microorganisms alter the behavior, distribution, and abundance of animal species, prey-predator interactions, and many other aspects of the ecosystems. Consequently, these interactions affect the ecology of pathogens and infectious diseases. Of note, diverse ecosystems are more resilient to changes and insults than more homogeneous environments. Thus, the preservation of biodiversity is associated with reduced changes in the ecology of hosts and their pathogens and, therefore, with a lower risk of infectious diseases emergence (Mills, 2006; Keesing *et al.*, 2010; French and Holmes, 2020; Gibb *et al.*, 2020; Mendoza *et al.*, 2020). Moreover, deforestation, mining, intensive land use, and other activities associated with the degradation of ecosystems, in connection with the previous topic approached in the present review, increase the interaction of humans with different wild species, facilitating spillover events. These activities also increase human exposure to vectors of different diseases, such as mosquitoes (Ellwanger *et al.*, 2020a).

Forests play a crucial role in regulating the global climate. The loss of vegetation cover is associated with higher levels of greenhouse gas emissions in the atmosphere (e.g., carbon dioxide), loss of water cycle regulation, and other environmental disturbances, fueling the rise in global average temperature and climate changes (Shukla *et al.*, 1990; Bonan, 2008; Fearnside, 2008; Nogueira *et al.*, 2018; West *et al.*, 2019). Host-pathogen interactions and vector dynamics are affected by many abiotic factors, such as wind and rain patterns and changes in temperature. Therefore, climate changes and extreme weather events are important determinants of the emergence and spread of infectious diseases. Extreme climatic events, such as heavy rains, hurricanes and floods, facilitate the proliferation of disease vectors and are associated with the increase in cases of diseases such as gastroenteritis, leptospirosis, and cholera. Besides that, higher temperatures and extreme weather events facilitate fires in forest environments, contributing to habitat loss and threatening several species (Wu F *et al.*, 2016; Ellwanger and Chies, 2018b; Khan *et al.*, 2019; French and Holmes, 2020).

Deforestation, biodiversity loss and climate change are highly connected and give rise to a vicious circle, favoring the spread of infectious diseases through many mechanisms. The frequency and intensity of outbreaks and epidemics tend to increase as climate changes become more intense. For these reasons, biodiversity preservation and reduction of climate change must receive special attention on the agenda focused on the control and prevention of infectious diseases (Ellwanger *et al.*, 2020a).

### Vector control

Animal vectors transmit over 17% of all infectious diseases in the world. Vector-borne diseases affect an enormous number of individuals worldwide in terms of morbidity and mortality, causing 700,000 deaths each year. Vector-borne diseases are caused by viruses (e.g., Dengue virus, Zika virus), bacteria (e.g., Rickettsial diseases, typhus), and parasites (e.g., lymphatic filariasis, malaria, schistosomiasis). Mosquitoes of the genus *Aedes*, *Anopheles* and *Culex*, along with ticks, triatomine bugs, aquatic snails, lice, fleas, tsetse flies, blackflies, and sandflies are the most relevant disease vectors. Mosquitoes alone are responsible for the transmission of various diseases, including chikungunya, dengue, lymphatic filariasis, yellow fever, Zika, Rift Valley fever, malaria, Japanese encephalitis, and West Nile fever. Of note, mosquitoes are amongst the animals that cause the highest rates of morbidity and mortality in the world (WHO, 2020a, b).

The control of vector-borne diseases is a fundamental action to reduce the global burden of infectious diseases. Vector control actions must be implemented at the local level but must be coordinated globally. According to the World Health Organization, effective vector control should include poverty reduction, promotion of good health and well-being, access to clean water and sanitation, development of sustainable cities and communities, climate actions, and partnerships between nations to promote global health (WHO, 2017b). Specifically, vector control involves a series of strategies that must be implemented according to the needs of each location, country and epidemiological particularities, which may involve the use of insecticides, predator species, habitat manipulation, house improvement, the release of genetically modified mosquitoes in the environment, among other strategies (Flores and O’Neill, 2018; Wilson *et al.*, 2020). Vector control is an investment that brings economic returns due to the prevention of numerous infectious disease cases, saving the lives of thousands of people and preventing consequential economic losses (Alonso *et al.*, 2017).

### Environmental sanitation

Adequate conditions of environmental (basic) sanitation are determinants of population health and include mainly the access to treated water, garbage collection, and sewage system and treatment. Along with vaccination, environmental sanitation is the factor that most contributed to the promotion of public health in the last century (Institute of Medicine, 1988; Nabel, 2013; Hodson, 2019). However, ~2.5 billion people in the world still do not have access to adequate sanitation conditions. Several diseases are associated with inadequate sanitation facilities and lack of access to treated water and sewage treatment, especially parasitic and viral enteric diseases (Mara *et al.*, 2010; Adane *et al.*, 2017; Freeman *et al.*, 2017). For instance, inadequate conditions of sanitation and poor hygiene practices are strongly associated with a higher incidence of childhood diarrhea in Brazil (Heller *et al.*, 2003; Bühler *et al.*, 2014); strikingly, it is estimated that 2,195 children die daily of diarrhea in the world, and 88% of these deaths are due to unsafe water, poor sanitation systems and insufficient hygiene (CDC, 2017). 

In addition to diseases transmitted by contaminated water, the lack of sanitation facilitates the proliferation of mosquitoes that spread numerous viral species (e.g., Dengue virus, Zika virus, West Nile virus). Accordingly, poor water supply services lead people to store water in containers, whereas the lack of adequate sewage systems gives rise to open sewers; these factors prove breeding sites for mosquitoes, contributing to an increase in vector-borne diseases (Degroote *et al.*, 2018). The proliferation of other urban pests that can transmit various pathogens to humans, such as rodents, is also favored by inadequate sanitation services (Moreira *et al.*, 2013; Rahelinirina *et al.*, 2019; Murray *et al.*, 2020). The diseases associated with the lack of sanitation also cause severe economic damages to the affected families and to the economy of the countries, since these diseases cause significant losses in working days and huge expenses with the treatment of the affected individuals, among other related issues (Van Minh and Hung, 2011; Fuente *et al.*, 2020). 

Of note, the lack of environmental sanitation is associated with 10% of the global human disease burden (Mara *et al.*, 2010). For these reasons, investments in sanitation could minimize numerous public health problems and bring robust economic gains (Hutton *et al.*, 2007; Van Minh and Hung, 2011; Prüss-Ustün *et al.*, 2017). Environmental sanitation is, therefore, one of the most effective and robust factors for the control and prevention of infectious diseases.

### Infectious disease surveillance

Surveillance is one of the most effective strategies for the control of infectious disease outbreaks and containment of emerging pathogens. Robust epidemiological surveillance systems allow the monitoring of the circulation of pathogens in humans, non-human animals, and human-animal interfaces, facilitating the early detection of emerging diseases with potential of causing epidemics and pandemics. Also, epidemiological surveillance is the mechanism that allows detecting sudden increases in cases of a particular disease. Currently, genome-based technologies are used in the rapid and highly accurate diagnosis and surveillance of infections. These technologies also allow the monitoring of outbreaks, epidemics, and pandemics accurately and practically in real time (Ellwanger *et al.*, 2017; Geoghegan and Holmes, 2017; Grady and Loman, 2018; Holmes *et al.*, 2018; Ellwanger *et al.*, 2019).

Classic or genome-based surveillance strategies must focus on populations most susceptible to infectious diseases. It is also necessary to give importance to the risks associated with emerging pathogens, prioritizing those with high public health relevance (e.g., high transmissibility among humans, high mortality). In addition, an adequate surveillance system must prioritize partnerships between research institutions and public health agencies, focusing on long-term investments, where the training of technical personnel, diagnostic capacity, and rapid responses to outbreaks occurs in an integrated, systemic and lasting manner. Professionals involved in research activities in outbreak situations must be committed to building (or improving) solid diagnostic, healthcare, and surveillance systems at the location where the outbreak occurred. This approach will enable research activities and, in a complementary way, assist in controlling future outbreaks with the help of trained local scientists and health professionals (Heymann *et al.*, 2016; Yozwiak *et al.*, 2016; Yamey *et al.*, 2017; Holmes *et al.*, 2018; Ellwanger *et al.*, 2019). 

Pathogens and epidemics do not respect national borders or political and cultural barriers. Flaws in the surveillance systems can make infectious disease outbreaks become global emergencies. Therefore, actions focused on the control of infectious diseases require health diplomacy and respect for global and local ethical issues, which depend on robust international partnerships and cooperation between global leaders (Bedford *et al.*, 2019). The COVID-19 pandemic illustrates the importance of both the epidemiological surveillance and the quick response to the identification and control of a new potential pandemic pathogen. In this context, the evaluation of outcomes and consequences, comparing the different measures taken by different countries around the world during the COVID-19 pandemic, will help us to establish (and learn) which approaches have given better results in such a situation. Nevertheless, independently of the approaches and measures taken, it is quite appropriate to say that epidemiological surveillance is one of the most critical initiatives for the control of infectious diseases.

### Urban planning (control of human agglomeration and adequate urbanization)

Historically, epidemics have arisen in human populations since the establishment of the first urban settlements. In other words, epidemics appear in human history along with the first villages and cities. Human agglomeration is an essential factor for human-to-human transmission of infectious diseases. This kind of transmission sustains outbreaks and epidemics of non-vector borne infections. In brief, the concentration of people in cities promotes the ideal conditions for the spread of diseases transmitted by direct contact (Wolfe *et al.*, 2007; Bañuls *et al.*, 2013).

Human agglomeration, unplanned urbanization and de-urbanization (neglected/abandoned urban areas) facilitate the emergence and spread of infectious diseases since these factors are associated with sustained human-to-human transmission of pathogens, lack of sanitation, closer contact with wildlife, garbage accumulation, and proliferation of disease vectors (Gubler, 2011; Neiderud, 2015; Herfst *et al.*, 2017; Dalziel *et al.*, 2018; Eskew and Olival, 2018; Tian *et al.*, 2018). Therefore, urban planning that prioritizes the population access to health infrastructure is a meaningful way to control infectious diseases, since it creates the conditions necessary for life in cities to be healthy, avoiding human settlements in environments without adequate sanitary conditions. For this objective to be achieved, it is crucial that architects, environmental and sanitary engineers, and urban planners actively participate in discussions involving the role of urban factors in the spread of infectious diseases, bringing solutions to mitigate these problems (Corburn, 2015; Giles-Corti *et al.*, 2016). In this context, it is important to keep in mind that not only the population size, but also human mobility patterns can contribute to the spread of infectious diseases. Taking the COVID-19 pandemic as an example, the rapid virus spread around the world was followed by different approaches aiming to mitigate or reduce its propagation, including non-pharmaceutical interventions, such as human mobility restrictions, school closures, and the establishment of social distancing measures. Of note, non-pharmaceutical interventions have significantly reduced the spread of the virus in several countries (Candido *et al.*, 2020; Flaxman *et al.*, 2020; Lai *et al.*, 2020; Pan *et al.*, 2020). We are now observing the figures and patterns of the different epidemic waves that are propagating from big to small cities, and from one country to another. Certainly, we will have much to learn about viral spread patterns in the next years by assessing, and questioning, the decisions taken during the period of this pandemic, and its consequences.

### Social actions to promote the reduction of population growth

The world population is close to 8 billion individuals and, currently, the number of people living in cities is higher than that of people living in rural regions (Buhaug and Urdal, 2013; Jensen and Creinin, 2020). The increase in the global human population causes a series of social, health, and environmental concerns. High birth rates in families with social and economic vulnerabilities are associated with poverty and lack of access to formal education, creating a vicious circle of social problems. Also, the growing demand for natural resources to meet human consumption requirements causes environmental degradation, leading to the collapse of ecosystems and reduction of biodiversity (Vörösmarty *et al.*, 2000; Allen, 2007; Crist *et al.*, 2017; Gomes da Silva and Gouveia, 2020). Overpopulation, poverty, high demand for food and consumer goods, large-scale agricultural practices and industrial production, and environmental degradation act synergistically, facilitating the emergence of new pathogens among human populations. The spread of known infectious diseases is also facilitated in overpopulated regions.

Therefore, overpopulation and its interconnected factors must be controlled as an additional way to prevent and contain the spread of infectious diseases. This action should be applied through poverty reduction, access to birth control advice and methods, and gender equality. These goals require investment in the promotion of formal education and improvements in access to contraceptive methods (Allen, 2007; Crist *et al.*, 2017; Jensen and Creinin, 2020). As a classical example, women’s educational attainment is the main factor involved in both the reduction of birth rates and delayed childbearing. Better knowledge regarding the use of contraceptives and more efficient access to these methods has contributed to the observed low fertility in higher social strata worldwide (Kravdal, 1992; Mathews and Ventura, 1997). 

Taking together, promoting social actions focused on the reduction of population growth is an additional way to control the spread of infectious diseases. However, it is important to highlight that mandatory governmental measures of birth control should be emphatically avoided.

### Sex education and promotion of safe sex

The promotion of safe sex is an essential initiative for the prevention of sexually transmitted infections and encompasses a series of joint actions, including campaigns focused on the prevention of these infections, the distribution of male and female condoms, promotion of sexual health through medical assistance, and school-based sexual education. In terms of public health, these actions have beneficial effects on the reduction of sexually transmitted infection rates. For these actions to be applied effectively, they must be demystified, viewed without prejudice or religious bias, being properly discussed and expanded, focusing on adolescents, adults, and the elderly of all sexual orientations (Mullinax *et al.*, 2017; Ford *et al.*, 2017).

In addition to the classic initiatives of promotion of safe sex, other innovative strategies with the same purpose should be encouraged, such as the Pre-Exposure Prophylaxis (PrEP), focused on the prevention of HIV infection (CDC, 2019). PrEP consists of taking anti-HIV drugs by uninfected individuals to prevent HIV infection in the case of exposure to the virus. Robust evidence indicates that PrEP is safe and highly effective, virtually eliminating the risk of infection if appropriately taken (Grant *et al.*, 2010; Fonner *et al.*, 2016; McCormack *et al.*, 2016). It should be offered mainly to individuals with risky behavior or with a history of repeated potential exposure to HIV. Together, sexual education and the various available forms of promoting safe sex have a significant impact on reducing the rates of sexually transmitted infections.

### Promotion of hygiene practices

In 1878, Louis Pasteur established that germs were causative agents of infectious diseases. After the “Germ Theory of Diseases” was accepted, it became evident that personal and familial hygiene practices were effective in reducing the occurrence of infections. The connection between hygiene practices (e.g., hand hygiene, use of soap, adequate waste disposal, hygiene education) as an effective way to avoid infections and reduce human mortality had been supported by robust and multiple evidence (Greene, 2001; Larson and Aiello, 2001; Aiello and Larson, 2002; Casanova and Abel, 2013). 

Although the pivotal role of personal hygiene practices as a strategy to prevent diseases is well established in high-income countries, this is still not the reality for many low-income nations due to the lack of knowledge or scarcity of structure for the distribution of treated water, sewage collection, and appropriate kitchens and bathrooms. To reduce the diseases associated with the lack of personal hygiene, governments need to ensure adequate housing conditions for the population, with proper sanitary installations, in addition to investing in education on how to prevent diseases linked to poor hygiene. In high-income countries, simple hygiene practices should be reinforced, such as hand washing after touching or manipulating structures for public use. These simple measures are of crucial importance to contain the spread of viral infections, especially in the months of seasonal pathogens’ circulation (Inaida *et al.*, 2016; WHO, 2020c). 

### Access to food, food security and proper nutrition

The nutritional status significantly influences the susceptibility and pathogenesis of infectious diseases in several ways. For example, protein deficiency and malnutrition increase the susceptibility to infections, and facilitate the pathogenesis of diseases, including HIV infection, malaria, and tuberculosis (Cegielski and McMurray, 2004; Schaible and Kaufmann, 2007; Katona and Katona-Apte, 2008; Ibrahim *et al.*, 2017). 

Recently, maternal protein deficiency was associated with increased susceptibility to congenital Zika syndrome, a condition characterized by developmental impairment in the fetus and caused by maternal Zika virus infection (Barbeito-Andrés *et al.*, 2020). This finding exemplifies how nutritional status can act as an important cofactor in the outcome of viral infections. Also, deficiency of micronutrients (e.g., vitamin A, selenium, iron) has critical impacts on immune responses to infectious diseases. For example, zinc deficiency was associated with increased morbidity of several infections, including malaria and respiratory infections, especially in children (Bahl *et al.*, 1998; Bhaskaram, 2002; Walker and Black, 2004; Ellwanger *et al.*, 2011). These examples indicate that proper nutrition is a public health issue that also reverberates on the prevention of infectious diseases.

Adequate eating habits depend on access to information and are influenced by cultural habits. However, these factors are just a few of the many determinants of proper nutrition. Importantly, proper nutrition is highly dependent on food security, which means not only access to food with guaranteed nutritional and microbiological quality: food security means that individuals and families would have enough income to acquire these foods sufficiently, in an accessible, constant and sustainable way (Schmidhuber and Tubiello, 2007; Glamann *et al.*, 2017). Many populations depend on bushmeat consumption to obtain sufficient intake of protein, which results in risks for the emergence of new human infectious diseases. Therefore, the promotion of food security is essential to prevent vulnerable populations depend on bushmeat as a source of protein. In other words, food security plays a very important role in reducing human contact with potential new pathogens from meat and biofluids of wild animals (Friant *et al.*, 2020). However, besides increasing food security and proper nutrition it is necessary to promote and maintain sustainability in agricultural practices (Sayer and Cassman, 2013). Innovative agricultural technologies and policies are needed to face environmental problems. For example, regenerative agriculture, permaculture and other sustainable agricultural practices must be developed and expanded, reducing energy demands and decreasing the environmental impacts of the food production chain (Rhodes, 2012; Akhtar *et al.*, 2016). Moreover, livestock production must meet animal hygiene and sanitation measures in order to guarantee not only animal health, but also food supply, food security and human health (Bianchini *et al.*, 2019).

 Food security, agricultural practices, environmental issues, and proper nutrition are connected and have important long- and short-term impacts on the environment, public health and the control and prevention of infectious diseases. Therefore, governments have a responsibility to guarantee these fundamental rights to their citizens. 

### Reduction of social inequalities

Social inequality is one of the main drivers of poor population health. Individuals with insufficient income are incapable of having proper daily diets, invest in education and professional training, and have adequate leisure activities as a way to promote physical health and reduce stress, being, therefore, more susceptible to several diseases. Of note, access to health services is essential for the diagnosis, treatment, and prevention of diseases. In an environment of social inequality, wealthier individuals have better access to these services. In contrast, most impoverished populations face many difficulties in accessing health services because they generally live far from the places where these services are offered and also have less access to means of transport. Other factors aggravate this scenario, such as the lack of formal education commonly observed in the poorest individuals, making it difficult for this portion of the population to get medical assistance (Farmer, 1996; Quinn and Kumar, 2014). 

Social inequalities and poverty have a direct and very palpable impact on infectious diseases. For example, the spread of the tuberculosis bacillus (*Mycobacterium tuberculosis*) is facilitated in poorly ventilated spaces and inadequate housing, factors commonly observed in the homes of the low-income populations, in which many people live together in small spaces (Lönnroth *et al.*, 2009; Lönnroth *et al.*, 2010; Lygizos *et al.*, 2013). Moreover, people in poverty live in homes often without access to safe water and other adequate conditions of environmental sanitation, allowing the occurrence of different enteric diseases, soil-transmitted helminth infections, Leishmaniasis, among other diseases (Cifuentes and Rodriguez, 2005; Hotez *et al.*, 2005; Alvar *et al.*, 2006; Echazú *et al.*, 2015). Low income, poor education, unemployment, discrimination, violence, and other poverty-related problems are associated with increased HIV infection burden and poor access to antiretroviral therapy (ILOAIDS, 2005; Kalichman *et al.*, 2006; Haacker and Birungi, 2018; Leddy *et al.*, 2019). Poor people are also more susceptible to the COVID-19 pandemic, as they live in conditions more prone to the spread of the SARS-CoV-2, in addition to being more susceptible to mortality due to this infection (Ahmed *et al.*, 2020). Taking together, the reduction of social inequalities is a robust form of prevention and control of infections at the population level.

## Impacts of host genetics on susceptibility to infections and disease progression: SARS-CoV-2 as a study model

### The role of host genetic on infectious diseases

Researchers have learned that when individuals are exposed to pathogens, some of them will be infected (normal or higher susceptibility to infection) whereas others will not be infected (reduced susceptibility or resistance to infection). Also, a remarkable feature observed in most infectious diseases is inter-individual phenotypic variability, with infected individuals presenting clinical features ranging from asymptomatic infection to fatal outcome during clinical progression. In many diseases, this clinical variability depends on immune responses against pathogens, which are influenced by host genetic factors (Mozzi *et al.*, 2018; Casanova and Abel, 2020).

Several infectious diseases follow a complex mode of inheritance, with multiple genetic and environmental factors contributing to susceptibility. Also, genetic variants have distinct effects on the phenotype, with major or modest effect (Stringer *et al.*, 2011). Classical examples of major contributor effects were found in malaria, in which natural erythrocyte polymorphisms are protective against severe malaria (Ruwende *et al.*, 1995; Allen *et al.*, 1997; Egan, 2018). Also, homozygous individuals for the 32 base-pair deletion in the *CCR5* gene (CCR5∆32) are highly resistant to HIV infection (Ellwanger *et al.*, 2020b). 

Human leukocyte antigen (HLA) alleles are the prototypical candidates for genetic susceptibility to infectious diseases since HLA molecules are responsible for presenting viral antigens to CD4^+^ and CD8^+^ T cells (Horton *et al.*, 2008). HIV, hepatitis C and B, leprosy, tuberculosis, leishmaniasis, helminthiasis, dengue and influenza are some examples of infections for which susceptibility or disease progression are influenced by specific HLA alleles (Blackwell *et al.*, 2009). 

As examples of HLA alleles associated to susceptibility and disease progression, HLA-B*27 and HLA-B*57 alleles were associated to slower progression to AIDS, control of viral load and high frequencies of CD4^+^ T cells (Migueles *et al.*, 2000; Chopera *et al.*, 2008); conversely, the HLA-B*35 allele was associated with rapid disease progression to AIDS (Tomiyama *et al.*, 1997). The HLA-A*11, HLA-B*35 and HLA-DRB1*10 alleles were associated with susceptibility to influenza H1N1 infection (Dutta *et al.*, 2018). The HLA-A*02:07 and HLA-B*51 alleles were associated with increased secondary severe disease caused by Dengue virus (Stephens *et al.*, 2002). Also, the HLA-DRB1*1202 allele was associated with susceptibility to SARS-CoV (Keicho *et al.*, 2009), and in contrast, HLA-Cw1502 and HLA-DR0301 alleles were associated with resistance against SARS-CoV (Wang *et al.*, 2011).

Our research group has already made several contributions regarding the role of host genetics on infectious diseases. For example, we have already investigated the impact of polymorphisms of the *TLR-9*, *HLA-G* and *CCR5* genes on viral hepatitis (Da Silva *et al.*, 2014; Valverde-Villegas *et al.*, 2017a; Ellwanger *et al.*, 2020c). These studies helped to understand, for example, which populations have differences in susceptibility to these infections. Also, the immune response to infections is influenced by interactions between different *loci*. Therefore, studies addressing epistatic interactions contribute to the understanding of the role of host genetic on infectious diseases. For example, some HLA alleles in combination with other gene variants were associated with delayed progression to AIDS (Magierowska *et al.*, 1999; Martin *et al.*, 2002), and a *CXCL10* rs56061981 and *CCL22* rs4359426 combination predicted susceptibility to HIV infection (Valverde-Villegas *et al.*, 2017b). 

Understanding the effects of human genetic polymorphisms can also assist the choice of treatments and therapies against infections. For example, the HLA-B*5701 allele is associated with hypersensitivity reaction to the anti-HIV drug Abacavir in certain ethnic groups (Mallal *et al.*, 2002, 2008); hence, genotyping of this allele is part of standard HIV treatment care in developed countries and represents a good example of how human genetic factors can significantly influence the treatment of a given infection.

### Host genetics and vaccination

Variable immune responses following immunization are linked, at least in part, to host genetic factors (Ellwanger and Chies, 2019). A study performed with West African population suggested that both HLA and non-HLA loci are responsible for the ability to respond to a vaccine (Newport *et al.*, 2004). Some remarkable examples were observed concerning measles and hepatitis B vaccination. Response failure to measles vaccine has an important pattern associated with heritability and family clustering (Jacobson *et al.*, 2003; Jacobson and Poland, 2004). Also, different studies have shown that both antibody levels in response to hepatitis B vaccine and vaccine responsiveness are highly influenced by host genetics factors (Kruskall *et al.*, 1992; Newport *et al.*, 2004; Höhler *et al.*, 2002). However, the impact of genetic features on vaccine immunogenicity is still a neglected topic (Ellwanger and Chies, 2019).

Genetic polymorphisms associated with protection from infectious diseases can also provide useful information for vaccine design through the strategy known as “reverse immunogenetics”. For example, HLA alleles strongly associated with a particular phenotype may be used in immunoinformatic studies aiming to identify the best epitopes for inclusion in a particular vaccine (Davenport and Hill, 1996).

### SARS-CoV-2 as a study model

SARS-CoV-2 is a novel single-stranded RNA betacoronavirus, which is responsible for the COVID-19 pandemic (Huang *et al.*, 2020). On October 16, 2020, the pandemic had affected more than 39 million individuals worldwide (Dong *et al.*, 2020; Johns Hopkins University, 2020). The COVID-19 has higher severity and mortality rates among elderly people and individuals with comorbidities, such as hypertension, diabetes, cardiovascular, and pulmonary diseases (Zhou F *et al.*, 2020). Approximately 80% of infected individuals are adults with mild symptoms (Cao *et al.*, 2020).

The main route of SARS-CoV-2 transmission is through respiratory droplets but individuals can also be infected through contact with surfaces and objects contaminated with the virus (Yang and Wang, 2020). To infect host cells, SARS-CoV-2 viral spike glycoprotein interacts with the Angiotensin-Converting Enzyme 2 (ACE2), which is expressed on the host cell membrane. Also, viral entry is mediated by the host cell serine protease TMPRSS2, which cleaves the viral spike protein, allowing the fusion of the viral particle and the host cell membrane (Hoffmann *et al.*, 2020). A study has reported a higher ACE2 expression in the lungs of individuals with comorbidities compared to control individuals, reinforcing that individuals with comorbidities have a higher risk to progress to severe COVID-19 (Pinto *et al.*, 2020). 

Looking at the involvement of cytokines in the pathogenesis of COVID-19, increased interleukin 6 (IL-6) levels are an important hallmark of severe COVID-19 cases, being associated with a higher risk of death (Chen *et al.*, 2020; Del Valle *et al.*, 2020; Herold *et al.*, 2020; Zhou F *et al.*, 2020). Deficient type I interferon (IFN) response was recently associated with an exacerbated inflammatory response in severe COVID-19 cases (Hadjadj *et al.*, 2020). On the other hand, there is robust evidence indicating that an increased IFN response would be a factor responsible for severe COVID-19 (Lee *et al.*, 2020; Lucas *et al.*, 2020; Perlman, 2020). Due to these contradictory results, the role of IFN is an important target for basic research and clinical trials (Lee and Shin, 2020).

The clinical course of SARS-CoV-2 infection was characterized into 3 phases: phase 1, an asymptomatic incubation period with or without detectable virus; phase 2, non-severe symptomatic clinical period with detectable virus; phase 3, severe respiratory symptomatic clinical period with high viral load. It has been hypothesized that during the early clinical phase 2, SARS-CoV-2 can cause non-severe symptoms and triggers protective immune responses. At this point of infection, the success of virus-eliminating immune response depends on the health status and genetic determinants potentially associated with susceptibility to SARS-CoV-2, such as HLA *loci* (Shi *et al.*, 2020; Wang *et al.*, 2020). 

Previous existent databases including human genomes, polymorphisms and transcriptomic data are being explored to understand the potential role of host genetic factors on the susceptibility to SARS-CoV-2 infection and COVID-19 progression. Importantly, previous clinical data collected from SARS-CoV-2-related coronaviruses, such a SARS-CoV, is helping in providing insights into the role of host genetics on COVID-19. A recent study using expression quantitative trait loci (eQTL) database analyzed the cumulative effect of genetic variants on ACE2 and TMPRSS2 expression in individuals from five different human populations (African, American, European, East Asian and South Asian) from the 1000 Genomes project (Ortiz-Fernández and Sawalha, 2020). This study suggested that genetic polymorphisms are associated with reduced ACE2 and TMPRSS2 expression in African populations. This phenotype might be associated with less susceptibility to SARS-CoV-2 infection in such populations. Moreover, this finding could help explain the inter-individual variability observed in the COVID-19 clinical progression (Ortiz-Fernández and Sawalha, 2020). However, according to the analyzes of the *ACE2* gene carried out by Fam *et al.* (2020), most human populations are similarly susceptible to SARS-CoV-2.

The potential influence of HLA alleles on SARS-CoV-2 infection is being assessed. The HLA-B*46:01 allele was previously associated with SARS-CoV infection severity (Lin *et al.*, 2003). In accordance, an *in silico* analysis found that HLA-B*46:01 allele predicted poor binding affinity for all SARS-CoV-2 peptides, suggesting an association of this allele with higher COVID-19 severity (Nguyen *et al.*, 2020). A genome-wide association study (GWAS) performed by The Severe Covid-19 GWAS Group found that the 3p21.31 gene cluster (*SLC6A20*, *LZTFL1*, *CCR9*, *FYCO1*, *CXCR6* and *XCR1*; lead variant rs11385942) is a susceptibility locus for COVID-19-related respiratory failure (Ellinghaus *et al.*, 2020). Furthermore, multiple evidence suggested that individuals with A blood group had higher risk of COVID-19 compared to individuals with non-A blood groups (Ellinghaus *et al.*, 2020; Wu Y *et al.*, 2020; Zhao *et al.*, 2020). Although these results need to be replicated, they corroborate a role for genetics in the different clinical outcomes of COVID-19.

The international COVID-19 Host Genetics Initiative (https://www.covid19hg.org/) was recently created with the objective to collect, share and analyze host genetic data to understand the role of genetic determinants of susceptibility to SARS-CoV-2 infection and COVID-19 progression. Of note, homogenized protocols for phenotype clinical classification is highly important in studies addressing the immunogenetic aspects of infectious diseases. Also, bioinformatics approaches used for the prediction of candidate targets for immune response against SARS-CoV-2 contribute to vaccine designs (Grifoni *et al.*, 2020). In this context, studies analyzing specific HLA alleles associated with induction of protective immune response to SARS-CoV-2 are needed.

In recent years, the knowledge regarding the impact of host genetic factors on infectious diseases was enriched mainly by studies addressing HIV/AIDS, HCV, and HBV. The knowledge accumulated through these studies will help to understand how host genetics influence the susceptibility to SARS-CoV-2 infection and COVID-19 progression. Specifically, such studies can provide information regarding 1) potential polymorphisms associated with susceptibility or resistance to SARS-CoV-2 infection; 2) the identification of host genetic factors that modulate COVID-19 progression; 3) putative epitopes for vaccine design; 4) insights into potential therapies and SARS-CoV-2 pharmacogenetics.

### Actions focused on mitigation of outbreaks and epidemics

In view of the public health risk posed by outbreaks, epidemics and pandemics, it is important that countries act together in undertaking measures to contain the spread of pathogens and mitigate diseases. Strategies such as travel restrictions, vaccination campaigns, surveillance and reporting of cases are examples of containment measures. When prevention and control measures are not met and an epidemic or pandemic event is established, it is important to focus on interventions to promote disease mitigation. The success of such measures relies on the participation of several stakeholders in all levels, including governments, institutions, communities and individuals. In addition, communication must be effective and clear in order to reach all levels of society, and hence broaden community awareness and participation (Schiavo *et al.*, 2014; Oppenheim *et al.*, 2018). 

Different interventions can be applied depending on the infectious disease and the stakeholder group where they are implemented. For example, mitigation measures for Dengue include epidemiological surveillance and vector control, which depends not only on actions developed by environmental and health authorities, but also on educational campaigns, individual awareness and participation of the community (Gregianini *et al.*, 2018). In the case of Yellow fever, for which a vaccine is available, vaccination campaigns and easy access to vaccines are effective prophylactic measures that depend on governmental authorities, healthcare settings and community acceptance. Another example of effective intervention to mitigate vector-borne disease at the community level is the use of insecticide-treated bednets in malaria-endemic areas (Yang *et al.*, 2018). 

The pandemic risk posed by new influenza viruses has led the World Health Organization to create strategies for the prevention, surveillance and control of influenza. As part of the Global Influenza Surveillance and Response System (GISRS), launched in 1952, initiatives such as the Global Influenza Program (GIP), the Pandemic Influenza Preparedness (PIP), and the Global Influenza Strategy 2019-2030 have been essential for epidemiological surveillance and development of diagnostic tools, vaccines and antivirals against influenza, as well as for building up laboratory capacity and capabilities to increase the number of tested individuals (Hay and McCauley, 2018; WHO, 2019). The benefits of these programs surpass influenza epidemics, since respiratory infections may also be caused by other viruses, some of which associated to severe disease, such as the highly pathogenic coronaviruses SARS-CoV, MERS-CoV and, more recently, SARS-CoV-2 (Guarner, 2020). 

The emergence of the SARS-CoV-2 at the end of 2019 was not dealt with equally by the authorities of different countries: while some governments promptly responded by closing borders, establishing lockdown and social distancing procedures, and testing individuals, others disregarded the severity and transmissibility of COVID-19. As a consequence, in few weeks COVID-19 had been declared a pandemic (WHO, 2020d) and, as previously mentioned, on October 16, 2020, more than 39 million cases of COVID-19 have been confirmed worldwide, including over 1 million deaths (Dong *et al.*, 2020; Johns Hopkins University, 2020). 

If on the one hand countries failed in containing the spread of SARS-CoV-2, on the other hand, due to the 2009 influenza pandemic, many countries were prepared for implementing mitigation actions. For example, in Brazil, reference laboratories that are part of the National Influenza Network were already equipped for laboratorial analyses of respiratory viral infection, hence were able to perform molecular analyses of samples from patients suspected of SARS-CoV-2 infection; with the advance of the disease in the country, other healthcare setting, including hospitals and private laboratories, also started to test individuals. In addition to governmental policies and community adoption of quarantine and social distancing behaviors, the world scientific community quickly responded to the emergence of the novel coronavirus by rapidly sequencing its genome, developing diagnostic kits, searching for drugs with antiviral activities against SARS-CoV-2 or able to alleviate COVID-19 symptoms.

Technological advances significantly contribute to the improvement of mitigation strategies. As mentioned, few days after SARS-CoV-2 was identified as the etiological agent of the severe pneumonia cases that had started at the end of 2019, scientists were able to sequence and analyze its genome, using deep sequencing and bioinformatic tools (Zhu *et al.*, 2020). Based on the genome sequence, diagnostic assays were promptly implemented and are being improved. 

Other international initiatives have been created for mitigating infectious diseases. Of note, the WIPO Re:Search Consortium was launched by the WHO and the World Intellectual Property Organization (WIPO) to accelerate the development of vaccines, diagnostic tools and drug research and development for neglected tropical diseases - mainly parasitic, bacterial and viral infections - through innovative research partnership and the sharing of knowledge and intellectual property among its members, which include universities and other scientific research institutions, as well as biotechnology and pharmaceuticals companies (WIPO, 2019). Such an initiative contributes to mitigating infectious diseases not only because it catalyzes biomedical and biotechnological innovations, but also because it engages scientists to get involved in projects to address unmet medical needs for some infectious diseases. 

Other mitigation strategies rely on advances in information technologies; for example, mobile phone data provides information about individual’s spatial behavior and mobility (Rubrichi *et al.*, 2018); health system databases and the internet contribute for notification of new cases, and higher computer capacity enables development of mathematical models to predict epidemic curves; mobile phones and other personal devices are also useful for notification of cases and healthcare support during social isolation. Finally, governmental actions to mitigate infectious diseases are important but have little impact without the acceptance and involvement of the community and other stakeholders. 

### Conservation of ecosystems as a public health strategy

There is an urgent need for an international, targeted, and well-coordinated action plan for the conservation of global ecosystems (Watson *et al.*, 2020). Actions for environmental preservation are mainly focused on the protection of threatened species and mitigation of climate change. We would like to add that prevention of infectious disease outbreaks is an additional benefit to conservation efforts. This strategy is well accepted by the scientific community but rarely used as an important argument in defense of environmental policies. Acknowledging this scenario can bring many benefits to society, and we urge all members of the scientific community to publicize and disseminate this information. In our opinion, awareness of the connections between ecosystems and the emergence/spread of infectious diseases will help to mobilize decision-makers and civil society in favor of the conservation agenda, especially considering the current scenario, with the ongoing COVID-19 pandemic.

In Brazil, government actions focused on the environment have been greatly weakened (Carvalho *et al.*, 2019; Ferrante and Fearnside, 2019; Hope, 2019). Currently, deforestation and other environmental disturbances in the Amazon region are growing (Artaxo, 2019; Escobar, 2019; INPE, 2020; Matricardi *et al.*, 2020), and attempts to facilitate the hunting of wild animals are on the rise (WWF, 2019). Deforestation and other environmental disturbances are associated with the emergence and spread of different infections (Ellwanger *et al.*, 2020a). Therefore, conservation efforts must be presented to everyone, from the lay public to specialized professionals and governments as concrete and effective public health measures, capable of avoiding cases of many vector-borne infections. 

Handling of bushmeat, for example, facilitates spillover events that can introduce new pathogens into the human population, as happened with HIV (Hahn *et al.*, 2000) and potentially with the current COVID-19 (Zhang and Holmes, 2020), representing an additional reason to limit hunting of wild animals. Of note, these points must be mentioned when drafting laws and regulations focused on the preservation of wild species. Otherwise, we should ask: What are we learning from these situations? Put in simple, conservation of ecosystems is a public health issue and scientists have a key role to play in disseminating this pivotal information. 

### Infectious diseases following natural disasters

Natural disasters with atmospheric, geological, and hydrological origins are disruptions of the ecological system that impact the harmonious relationship of human societies with the environment and other species (Lemonick, 2011). Disasters include drought, landslides, earthquakes, volcanic eruptions, floods, hurricanes, and tsunamis, and they have serious social, economic and health consequences (Watson *et al.*, 2007). Disaster-preparedness systems are important in order to reduce the public health impact during and following a disaster; however, many low- and middle-income communities living in areas more susceptible to natural disasters lack infrastructure, resources and disaster-response plans (Lemonick, 2011). In addition, poverty and social inequality, as well as environmental degradation and rapid population growth are characteristics of vulnerable communities that contribute for disaster severity. Of note, most deaths associated with natural disasters are due to fractures, burn, blunt trauma, drowning and crush-related injuries; communicable diseases after natural disasters are less common and are usually associated with after-effects of the disaster, which includes environmental changes, population displacement, proliferation of (and exposure to) disease vectors, poor water and sanitation conditions, poor food supply, limited access to healthcare services, lack of health professionals and other human resources (Watson *et al.*, 2007; Kouadio *et al.*, 2012). 

 The infectious diseases that most commonly occur during or after a natural disaster are diarrhea, acute respiratory infections, measles, cholera, and leptospirosis. Of note, crowding due to population displacement during a disaster contribute for the spread of diseases that are transmitted person-to-person by airborne droplets, such as respiratory infections - both bacterial (e.g., tuberculosis, pertussis, Legionella) and viral (e.g., influenza, respiratory syncytial virus, measles, coronaviruses, and others) (Lemonick, 2011).

During the occurrence of a disaster and the first 0-4 days that follow it, emergency services are the most required. Approximately 4 weeks after the disaster, during the recovery phase, there is an increased risk for the occurrence of diseases with a longer incubation period, as well as vector-borne diseases and chronic diseases; hence, surveillance strategies are important for the prevention of outbreaks, epidemics or, in a worst case scenario, pandemics. Examples are outbreaks of disease resulting from the displacement of people in overcrowded fields and cross-contamination of water sources with fecal material and toxic products (Lemonick, 2011; Kouadio *et al.*, 2012).

As above-mentioned, one of the main factors that aggravate these outbreaks is socio-environmental vulnerability. For example, every year intense precipitation causes floods and land sliding in different regions of Brazil. Accordingly, in 2013 the state of Acre, located in Northern Brazil and that has a low human development index, experienced serious rainfalls that were followed by outbreaks of leptospirosis; whereas in the Southern state of Santa Catarina, which has a high human development index and also suffered severe rainfalls in 2013, cases of leptospirosis did not increase significantly (S2iD, 2013). 

Another example is the cholera outbreak in Haiti that occurred 10 months after the 2010 earthquake. While approximately 200,000 people died in the disaster, 665,000 cases of cholera were reported, with 8,183 deaths (CDC, 2014). Notably, the country was collapsed by the earthquake: public health services structures had been reduced by damage and loss, there was a lack in infrastructure and sanitation systems, and work teams were still focused on caring for victims - the perfect storm for the cholera epidemic (Fraser, 2010; Gelting *et al.*, 2013).

These examples show that, when planning for the response to natural disasters, emergency services must define the strategies to monitor and contain disease outbreaks that arise weeks after the disaster. Such actions require post-disaster surveillance systems able to quickly detect cases of epidemic-prone diseases (Watson *et al.* 2007). If not contained, an outbreak that follows a disaster can turn a local problem into a regional or national epidemic. Moreover, developed countries should support developing countries in these strategies to respond to natural disasters that include the supply of vaccines, medical supplies, training of health professionals and efficient health surveillance systems.

### Translational ecology: practical actions, economic fund and political engagement for the control and prevention of infectious diseases

A higher number of people must recognize the preservation of biodiversity and ecosystems as an effective strategy for the prevention and control of infectious diseases. This strategy must be spread among the lay population and scientists from various fields. However, this strategy will only have practical results if applied in a systematic and lasting way, considering medical, economic and social aspects of the populations. Therefore, translational ecology is needed: knowledge and scientific data must be transformed into public policies and laws focused on the protection of biodiversity and ecosystems (Schlesinger, 2010; Enquist *et al.*, 2017; Sokolow *et al.*, 2019; Ellwanger *et al.*, 2020a; Xiao and Torok, 2020). Engaging politicians and decision-makers in discussions about the influence of the environment on the dynamics of infectious diseases is essential for translational ecology to be part of the political agenda of government agencies at local and national levels.

Creating economic strategies to deal with public health emergencies is also essential. In this sense, high-income countries should create a stock capital fund to help poor countries tackle emerging epidemics. Besides the humanitarian issue *per se*, by helping to mitigate or prevent the emergence of epidemics in less favored nations, high-income countries end up preventing such epidemics from reaching their territories, avoiding the socioeconomic impacts generated in a context of epidemic or pandemic. In fact, societal initiatives have already been created, but they are difficult to sustain during “inter-pandemic” periods. Furthermore, in addition to creating an investment fund to prevent epidemics and pandemics, it is extremely important to know where and how these financial resources should be applied (Yamey *et al.*, 2017; Berry *et al.*, 2018). 

Investments in the prevention of outbreaks and epidemics encompass hospitals, lab facilities and equipment, vehicles, surveillance networks, knowledge, and human capital. Such investments represent an economically advantageous strategy to follow, especially when compared to the high costs to mitigate the effects of epidemics and pandemics. Considering the current issues regarding climate change and biodiversity loss, outbreaks and epidemics are becoming increasingly common, especially in tropical countries. For these reasons, countries should use “inter-epidemic” periods to prepare themselves economically to deal with epidemic and pandemic periods (Morse *et al.*, 2012; Berry *et al.*, 2018; Dobson *et al.*, 2020).

The scenario mentioned above reinforces the need for international cooperation to avoid and deal with emerging infectious diseases. International funds focused on the prevention and control of infectious diseases is an urgent necessity (Ross *et al.*, 2015; Dobson *et al.*, 2020; Jia and Wu, 2020). In addition, scientific evidence showing the association of biodiversity loss and climate change with outbreaks and epidemics needs to be transformed into public policies focused on protecting the environment.

## Final considerations

**Figure 2 -:**
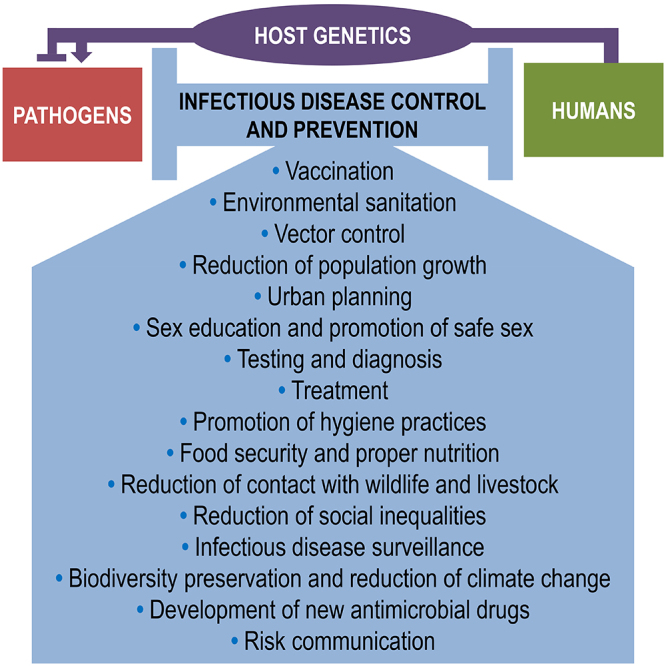
Actions for infectious disease control and prevention. The interactions between humans and pathogens can be facilitated or avoided depending on the presence or absence of actions of control and prevention of infectious diseases. Moreover, host genetics affects host-pathogen interactions: for example, HLA alleles and genetic polymorphisms can increase or decrease susceptibility to infectious diseases. These genetic traits also affect the progression of infectious diseases. This figure is based on references and discussions mentioned throughout the article.

The multiple interactions between humans and pathogens can be facilitated or avoided depending on the presence or absence of actions for the control and prevention of infectious diseases. Host genetic factors also interfere with host-pathogen interactions, since the genetic background modifies susceptibility to infections and disease progression (Figure 2). Of note, this article highlights that the effects of human genetics on the susceptibility and progression of infectious diseases are expressed within a complex context, where biological and social factors are playing important roles in the disease.

Robust evidence shows that the initiatives here addressed are crucial for the control and prevention of human infectious diseases. Scientists have a fundamental role in the search for alternatives to improve these initiatives. These professionals must also act on the dissemination of the importance of these actions, especially in the most vulnerable populations and regions. 

Considering the multiplicity of the actions for the control and prevention of infectious diseases, it is necessary to engage professionals from different fields. For example, biologists may commit to the development of new vector control tools and also be involved in the detection of host genetic factor associated with infectious disease phenotypes; veterinarians can contribute for surveillance of pathogenic agents such as protozoa, virus and bacteria in wild and domestic animals; social scientists can act on the identification of populations most vulnerable to infections; geneticists and molecular biologists can improve methods for pathogen detection; and medical scientists can develop better therapies. In other words, the control of infectious diseases must be performed within the One Health perspective, considering human, animal, and environmental drivers of infections. The boundaries between humans and non-human animals must be prioritized, as they are critical for the emergence of new human diseases. Therefore, epidemiological surveillance actions involving human-animal interactions and sanitary control in meat markets are essential for the prevention and control of new infectious diseases.

The application of the actions discussed here requires a significant amount of financial resources and the involvement of civil society and government institutions. To achieve these goals, the participation of politicians committed to public policies focused on the environment and health promotion is fundamental. The lack of connection between scientific research and public policies is the reason that often explains why there are flaws in the control of infectious diseases, even though there are already prevention and treatment strategies for many of them.

Finally, this article has pointed out the best directions that researchers and decision-makers committed with the control of infectious diseases must follow to perform research activities and promote health among society in a realistic and evidence-based way. Properly selecting the areas and initiatives that best fit the aspirations of researchers and the needs of each population help to optimize the financial resources for infectious disease control initiatives and research focused on pathogens, especially in developing countries where the resources are limited.
